# Abnormal brain functional connectivity leads to impaired mood and cognition in hyperthyroidism: a resting-state functional MRI study

**DOI:** 10.18632/oncotarget.14060

**Published:** 2016-12-21

**Authors:** Ling Li, Mengmeng Zhi, Zhenghua Hou, Yuqun Zhang, Yingying Yue, Yonggui Yuan

**Affiliations:** ^1^ Department of Endocrinology, Affiliated ZhongDa Hospital, School of Medicine, Southeast University, Nanjing, Jiangsu, 210009, China; ^2^ Department of Psychosomatics and Psychiatry, Affiliated ZhongDa Hospital, School of Medicine, Southeast University, Nanjing, Jiangsu, 210009, China; ^3^ Neuropsychiatric Institute, Affiliated ZhongDa Hospital, Southeast University, Nanjing, Jiangsu, 210009, China

**Keywords:** hyperthyroidism, degree centrality, functional connectivity, emotion, cognition

## Abstract

Patients with hyperthyroidism frequently have neuropsychiatric complaints such as lack of concentration, poor memory, depression, anxiety, nervousness, and irritability, suggesting brain dysfunction. However, the underlying process of these symptoms remains unclear. Using resting-state functional magnetic resonance imaging (rs-fMRI), we depicted the altered graph theoretical metric degree centrality (DC) and seed-based resting-state functional connectivity (FC) in 33 hyperthyroid patients relative to 33 healthy controls. The peak points of significantly altered DC between the two groups were defined as the seed regions to calculate FC to the whole brain. Then, partial correlation analyses were performed between abnormal DC, FC and neuropsychological performances, as well as some clinical indexes. The decreased intrinsic functional connectivity in the posterior lobe of cerebellum (PLC) and medial frontal gyrus (MeFG), as well as the abnormal seed-based FC anchored in default mode network (DMN), attention network, visual network and cognitive network in this study, possibly constitutes the latent mechanism for emotional and cognitive changes in hyperthyroidism, including anxiety and impaired processing speed.

## INTRODUCTION

Hyperthyroidism is one of the most common endocrine diseases. It is featured with excessive serum free triiodothyronine (FT3), free thyroxine (FT4) and suppressed thyroid stimulating hormone (TSH). As the recent studies have reported, hyperthyroidism is associated with some dysfunction of emotion and cognition such as nervousness, irritability, tremulousness, depression, anxiety, memory impairment, lack of concentration, declined executive function and so on [1–5].

However, the underlying mechanism of these neuropsychiatric alterations remains ambiguous. Multiple lines of neurobiological researches have revealed the existence of metabolic, morphological and functional brain alterations associated with hyperthyroidism. Based on positron emission tomography, metabolic changes of the brain in hyperthyroid patients have been found. Relative to healthy controls, patients with hyperthyroidism had reduced glucose metabolism in the frontal, limbic and temporal lobes [6, 7]. Besides, Zhang et al. [8] and Liu et al. [9] demonstrated a significantly decreased concentration of glutamate in the posterior cingulate cortex (PCC) in the hyperthyroidism group, indicating an underlying role of glutamate in brain dysfunction. On the other hand, structural variations of some brain regions have also been discovered in some studies applying resting-state functional magnetic resonance imaging (rs-fMRI). Zhang et al. [10] reported bilateral hippocampal atrophy in individuals with hyperthyroidism, as well as negative correlations between hippocampal gray matter volume and some clinical severity indexes. Another rs-fMRI study has observed an increase of gray matter volume in the right posterior lobe of cerebellum (PLC), as well as a decrease of gray matter volume in bilateral visual cortex and anterior cerebellum in hyperthyroidism compared to the euthyroid condition. These changes of gray matter volume are associated with sensorimotor functions and working memory [11], and indicate the cerebellum's important role in hyperthyroidism. In addition, there are some resting-state functional connectivity (FC) studies showing aberrant functional connectivity of the brain in hyperthyroid patients. Zhang et al. [12] defined the hippocampus as the seed region revealed decreased functional connectivity to bilateral anterior cingulate cortex (ACC), PCC and right medial orbitofrontal cortex. And the strength of functional connectivity was correlated with both the disease duration and depression level of hyperthyroidism. Furthermore, Gottlich et al. [13] recruited twenty-nine healthy men and gave them 8 weeks of oral administration of 250 μg levothyroxine per day to induce short-term hyperthyroidism. The study showed that short-term hyperthyroidism resulted in increased FC between temporal lobes and cognitive control network, indicating a key role of thyroid hormones in regulating paralimbic structures. These existing studies consistently suggested that impaired neurocognitive performances of hyperthyroid patients might be associated with abnormal alterations mainly in brain regions of default mode network (DMN), cognitive network as well as cerebellum. Based on the studies above, we speculate that hyperthyroid patients may exhibit some altered brain functional connectivities which constitute the neural substrate of emotional and cognitive dysfunction.

However, none of these approaches fully characterize the brain's functional connectome. While the seed-based method can be used to map the functional connectome in its entirety, it only provides a series of relationships between any given region and all other regions without taking the full pattern of connections into account. Recently, graph theory-based network analyses have been applied to explore brain connectivity within the whole-brain network [14–16]. In particular, degree centrality (DC) is a class of graph-theoretic measures assessing the importance of each node in brain network, in terms of its connectivity strength to every voxel [17–19]. In contrast to traditional seeding approaches that need to select a particular region based on priori hypothesis, this voxel-based whole-brain correlation analysis could provide the opportunity for an unbiased general search of abnormalities within the entire connectivity matrix of full-brain functional connectome [20, 21]. DC calculation has been widely used to uncover mechanisms of other psychosomatic disorders, such as Alzheimer's disease [22], Schizophrenia [23], Parkinson's disease [24], obsessive compulsive disorder [25] and major depressive disorder [26]. In order to provide a new insight for mechanism detection of neurocognitive and emotional impairment in hyperthyroidism, we performed DC analysis to identify voxels that showed altered FC with other voxels. Then, we conducted further FC analyses using the brain regions that showed significant alterations in the DC analysis, to obtain detailed information regarding the connectivity between a voxel and the particular regions that were changed. So we want to use DC and FC analysis to explore the underlying mechanism of the neuropsychological impairments in hyperthyroidism.

## RESULTS

### Demographic and neuropsychological results

Demographics and neuropsychological data for hyperthyroid patients and healthy subjects were summarized in Table 1. No significant between-group differences were found in age, education and gender. Compared with HC group, hyperthyroid group got significantly higher scores in HDRS (*P* < 0.001) and HARS (*P* < 0.001), but lower scores in executive function (*P* = 0.011) and visuospatial skills (*P* < 0.001). The raw scores of neuropsychological tests were shown in Supplementary Table S1.

**Table 1:** Demographic, clinical characteristics, mood and cognitive performances

Characteristic	Hyperthyroidism	Control	*p*-value
n	33	33	
Age (years)	37.36 ± 12.43	39.03 ± 13.28	0.600^a^
Female, n (%)	26.00(78.79)	26.00(78.79)	1.000^b^
Education levels (years)	13.24 ± 3.82	13.70 ± 4.06	0.641^a^
BMI (kg/m^2^)	21.61 ± 2.81	23.35 ± 2.55	0.011^a^
FT3 (pg/ml)	14.17(8.98−23.59)	3.02(2.77−3.33)	< 0.001^c^
TGAb (IU/mL)	269.00(25.07−540.60)	18.95(16.10−37.28)	< 0.001
TPOAb (IU/mL)	221.20(37.52−493.10)	14.84(11.75−19.29)	< 0.001^c^
Drug use, n (%)	15.00(45.45)	−	−
Disease duration (months)	9.94 ± 17.31	−	−
Neuropsychological test data (z-score)			
Mood			
HDRS^d^	0.70 ± 0.97	−0.70 ± 0.25	< 0.001^a^
HARS^d^	0.78 ± 0.85	−0.78 ± 0.23	< 0.001^a^
Processing Speed	−0.17 ± 0.89	0.17 ± 0.82	0.115^a^
Stroop Color^d^	−0.15 ± 1.09	0.15 ± 0.89	0.216^a^
Stroop Word^d^	−0.17 ± 1.02	0.17 ± 0.97	0.174^a^
TMT-A^d^	−0.18 ± 1.00	0.18 ± 0.98	0.140^a^
DSST^d^	−0.17 ± 1.00	0.17 ± 0.98	0.169^a^
Executive Function	−0.24 ± 0.76	0.24 ± 0.71	0.011^a^
Stroop Inhibition^d^	−0.03 ± 0.97	0.03 ± 1.04	0.807^a^
TMT-B^d^	−0.25 ± 0.81	0.25 ± 1.12	0.042^a^
DST^d^	−0.30 ± 0.99	0.30 ± 0.93	0.014^a^
VFT-1^d^	−0.34 ± 1.02	0.34 ± 0.87	0.005^a^
VFT-2^d^	−0.27 ± 1.13	0.27 ± 0.78	0.026^a^
Visuospatial Skills	−0.42 ± 0.92	0.42 ± 0.43	< 0.001^a^
CFT^d^	−0.33 ± 1.27	0.33 ± 0.44	0.007^a^
CDT^d^	−0.51 ± 0.97	0.51 ± 0.74	< 0.001^a^
Episodic Memory	−0.19 ± 0.87	0.19 ± 0.86	0.087^a^
AVLT-DR^d^	−0.06 ± 0.97	0.06 ± 1.04	0.611^a^
CFT-DR^d^	−0.31 ± 1.02	0.31 ± 0.89	0.011^a^

Abbreviations: BMI = Body Mass Index, FT3 = Free Triiodothyronine, TGAb = Thyroglobulin Antibody, TPOAb = Thyroid Peroxidase Antibody, HDRS = Hamilton Depression Rating Scale, HARS = Hamilton Anxiety Rating Scale, Stroop Color Total Time = Color naming subtest scaled score, Stroop Word Total Time = Word naming subtest scaled score, Stroop Inhibition Time = inhibition subtest scaled score; TMT = Trail-Making Test, DSST = Digit Symbol Substitution Test, DST = digit span test, VFT = Verbal Fluency Test, CDT = clock-drawing test, CFT = Rey-Osterrieth complex figure test, CFT-DR = Rey-Osterrieth complex figure test delayed recall, AVLT-DR = auditory verbal learning test–delayed recall.

^a^Two independent sample *t*-test.

^b^Chi-square test.

^c^Mann-Whitney rank test.

^d^Scale scores were transformed to standard Z values in order to avoid the influence of the different measurement units.

### DC analysis

**Figure 1:**
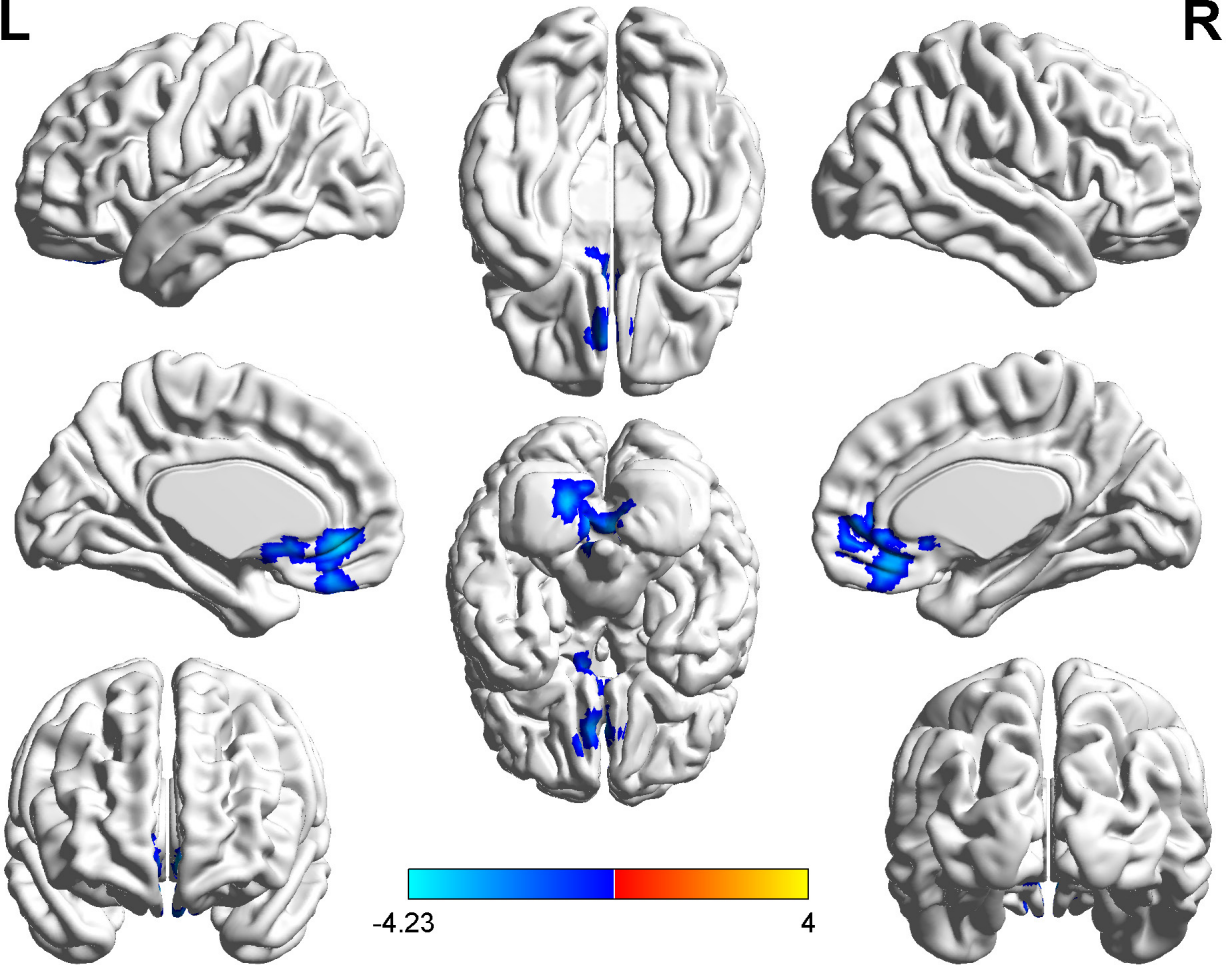
Significantly decreased (blue) DC in hyperthyroid patients (*P* < 0.05, AlphaSim corrected). The color bar indicates the T value from two-sample *t* test between hyperthyroidism and healthy control group.

Results of the two-sample *t*-test showed significant DC alterations for several related brain regions in hyperthyroid patients compared with healthy controls (P < 0.05, AlphaSim corrected; Table 2). We discovered that patients with hyperthyroidism had a decreased DC value, with a peak difference in left posterior lobe of cerebellum (PLC) and bilateral medial frontal gyrus (MeFG) (Figure 1). The peak points of these brain regions were then selected as seeds in further resting-state FC analyses.

**Table 2:** Brain regions showing significantly different DC between HPs and HCs

		BA	Peak area	MNI coordinates			Voxels number	Peak t value
				X	Y	Z		
DC	HPs < HCs		Left PLC	−15	−66	−48	143	−3.50
		32/9	Bilateral MeFG	−6	39	−6	289	−4.23

Note: Threshold was set at *P* < 0.05 (AlphaSim-corrected). X, Y, Z, coordinates of primary peak locations in the MNI space.

Abbreviations: MNI, Montreal Neurological Institute space; DC, degree centrality; HPs, hyperthyroid patients; HCs, health controls; BA, Brodmann area; PLC, cerebellum posterior lobe; MeFG, medial frontal gyrus.

### Seed-based functional connectivity analysis

**Figure 2:**
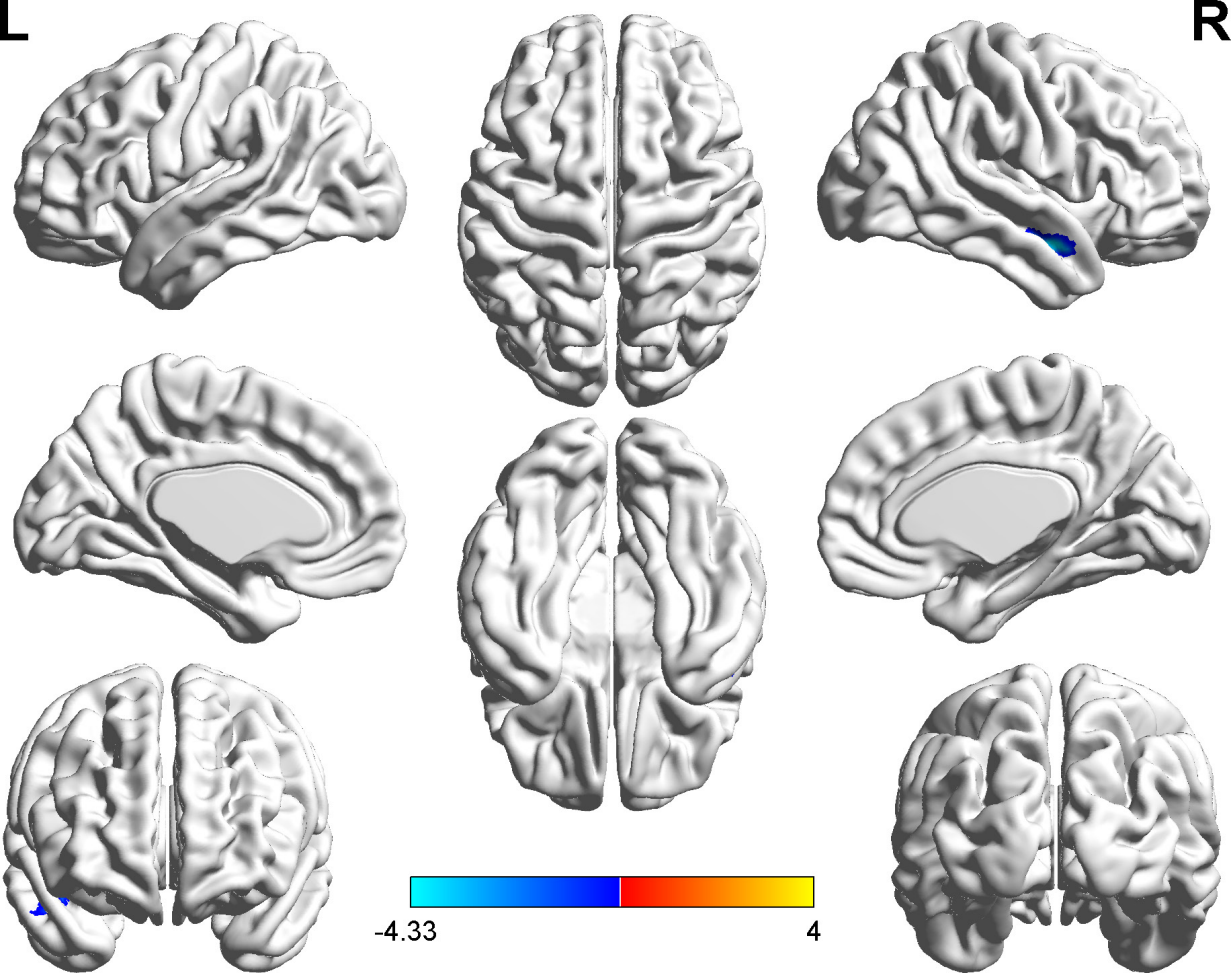
Brain regions showing decreased functional connectivity (blue) with the seed-1 point (−15, −66, −48) in the hyperthyroid group compared with the control group (P < 0.001, AlphaSim corrected) The color bar indicates the *T* value from two-sample *t* test between hyperthyroidism and healthy control group.

**Figure 3:**
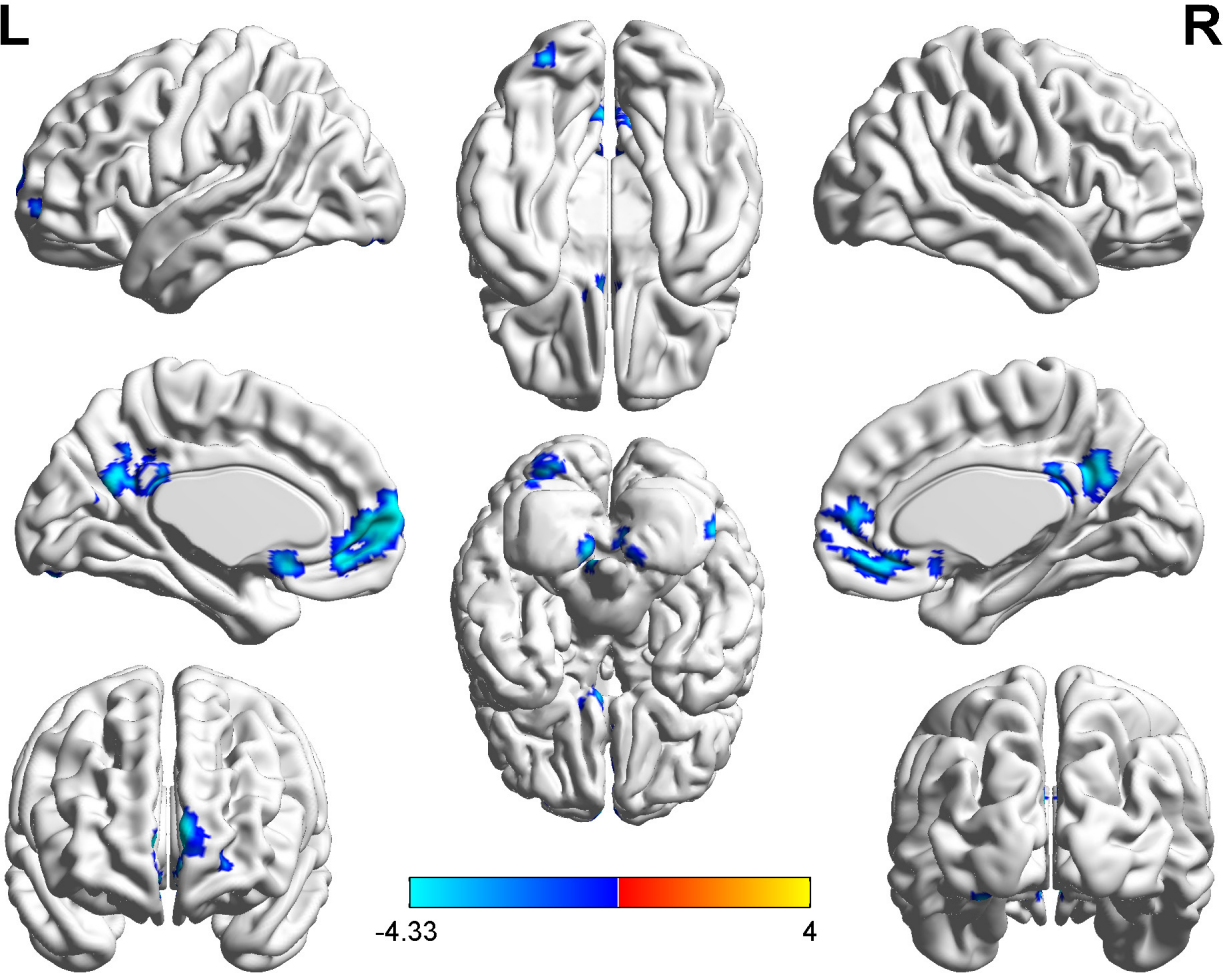
Brain regions showing decreased functional connectivity (blue) with the seed-2 point (−6, 39, −6) in the hyperthyroid group compared with the control group (P < 0.001, AlphaSim corrected) The color bar indicates the *T* value from two-sample *t* test between hyperthyroidism and healthy control group.

With the abnormal DC regions as seed points, the two-sample *t*-tests revealed significantly altered FC between seed regions and the whole brain when comparing hyperthyroid patients and healthy controls (Table 3, Figures 2, 3).

**Table 3:** Brain regions showing significantly different functional connectivity between HPs and HCs

Connected regions	BA	Peak area	MNI coordinates			Voxels number	Peak t value
			X	Y	Z		
Seed point (−15, −66, −48)							
	21	Right MTG	48	0	−18	23	−4.33
Seed point (−6, 39, −6)							
		Left PLC	−6	−51	−51	61	−5.07
		Right cerebellum	54	−63	−36	22	−5.06
	19	Left IOG	−27	−87	−21	44	−5.17
		Left caudate	−15	21	3	22	−4.13
	7	Bilateral PCu	−3	−63	24	60	−4.31
	9	Bilateral MeFG	−6	42	−9	243	−5.80

Note: Threshold was set at *P* < 0.001(AlphaSim-corrected). X, Y, Z, coordinates of primary peak locations in the MNI space.

Abbreviations: MNI, Montreal Neurological Institute space; HPs, hyperthyroid patients; HCs, health controls; BA, Brodmann area; MTG, middle temporal gyrus; PLC, posterior lobe of cerebellum; IOG, inferior occipital gyrus; PCu, precuneus; MeFG, medial frontal gyrus.

With the peak point (−15, −66, −48) of the left PLC as the seed-1 region, we found a reduced FC strength between seed-1 and the right middle temporal gyrus (MTG) in hyperthyroidism group compared to the control group (Figure 2). With the peak point (-6, 39, -6) of the bilateral MeFG as the seed region, we found a reduced FC strength of seed-2 to the left PLC, right cerebellum, left inferior occipital gyrus (IOG), left caudate, bilateral precuneus (PCu) and bilateral MeFG in hyperthyroidism group compared to the control group (Figure 3).

### Correlation analysis within the hyperthyroidism group

**Figure 4:**
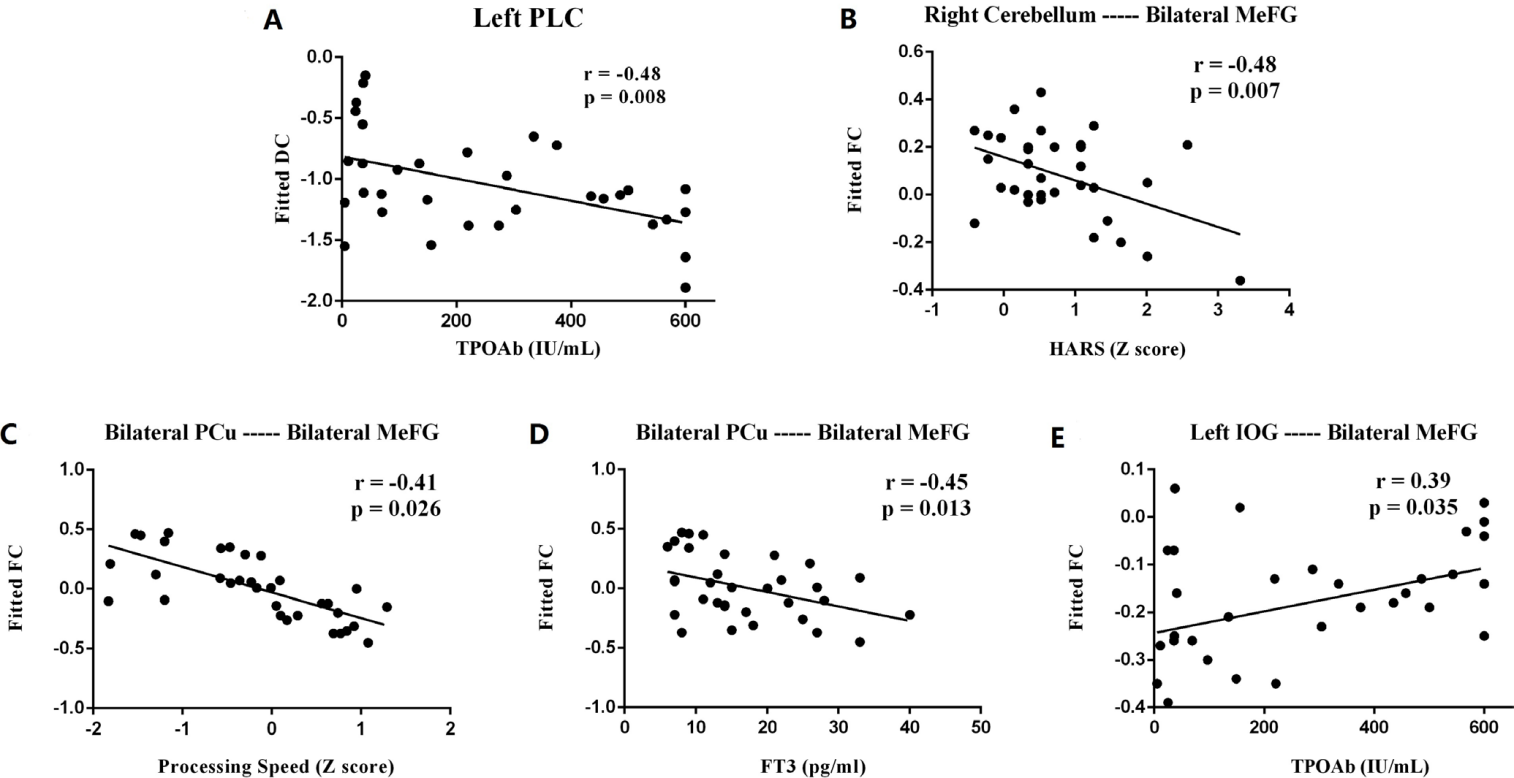
Scatter diagrams show the significant correlations between the psychological assessment z-scores, clinical indexes and values of DC, FC in hyperthyroidism group (**A**) DC in left PLC was in negative correlation with serum TPOAb (r = −0.48, *P* = 0.008); (**B**) FC between right cerebellum and MeFG was negatively correlated with HARS z scores (r = −0.48, *P* = 0.007); (**C**) FC between bilateral PCu and MeFG was negatively with processing speed z scores (r = −0.41, *P* = 0.026); (**D**) FC between bilateral PCu and MeFG was negatively with FT3 levels (r = −0.45, *P* = 0.013); (**E**) FC between left IOG and MeFG was positively correlated to TPOAb levels (r = 0.39, *P* = 0.035). HARS = Hamilton Anxiety Rating Scale, DC = Degree Centrality; FC = Functional Connectivity; PLC = Posterior Lobe of Cerebellum; MeFG = Medial Frontal Gyrus; PCu = Precuneus; IOG = Inferior Occipital Gyrus; r = Spearman's correlation coefficient.

After controlling for age, gender and years of education, partial correlation analyses were applied within the hyperthyroidism group. The reduced DC values of the left PLC were negatively correlated with serum thyroid peroxidase antibody (TPOAb) levels (r = −0.48, *P* = 0.008). And the decreased FC between right cerebellum and MeFG was negatively correlated with HARS Z scores (r = −0.48, *P* = 0.007). Besides, the decreased FC between bilateral PCu and MeFG was negatively with both processing speed Z scores (r = −0.41, *P* = 0.026) and FT3 levels (r = −0.45, *P* = 0.013). Finally, the reduced FC between left IOG and MeFG was positively correlated to serum TPOAb levels (r = 0.39, *P* = 0.035) (Figure 4).

## DISCUSSION

In the current study, we firstly investigated the primary hyperthyroidism-related intrinsic dysconnectivity of the whole brain functional networks by a combination of DC at the voxel level and seed-based resting-state FC analyses. This study reveals that hyperthyroid patients had decreased DC values in the left posterior lobe of cerebellum and bilateral medial frontal gyrus, displaying reductions of the number of direct connections within the whole brain networks in hyperthyroidism. The further seed-based FC analyses described more details about abnormal functional networks anchored in these regions mainly distributing in default-mode network, attention network, visual network and cognitive network.

Hyperthyroid subjects exhibited significantly lower DC in left PLC. This complements the prior VBM study which showed altered gray matter in bilateral cerebellum in hyperthyroidism [11]. Resting-state FC analyses showed a decreased functional connectivity between seed-1 located in left PLC and right MTG in the attention network. Meanwhile, lowered functional connectivity from both left PLC and right cerebellum to MeFG was detected in the present study. The cerebellum has long been considered to play a vital role in motor control [27, 28]. In addition to motor control, resting-state FC data has revealed that the cerebellum is part of cognitive networks with prefrontal and parietal associated cortices via cerebello-thalamo-cortical and cortical-ponto-cerebellar loops [29–32]. Moreover, it was reported that disrupted connectivity between cerebellum and middle temporal motion complex of dorsal attention network was associated with impaired cognitive function [35, 36]. Hence in the present study, the diminished functional connectivity anchored in the cerebellum possibly led to relevant cerebellum-supported cognitive impairments. Meanwhile, we discovered a negative correlation between right cerebellum-MeFG FC and HARS Z scores. Some studies regarding anxiety disorders including post-traumatic stress disorder [37] and bipolar disorder [38], have demonstrated that cerebellum is involved in the regulation of anxiety. Subsequent chronic cerebellar stimulation for neurologic disorders can yield decreased anxiety and improved mood [37]. So the functional connectivity between cerebellum and DMN may be associated with anxiety symptom. The underlying mechanism of the cerebellum dysfunction by the thyrotoxicosis is probably related to the expression of thyroid hormone receptor TRa1 and TRb1 in Purkinje cells in the cerebellum [38]. A study on hypothyroid mice demonstrated that, in the absence of thyroid hormones, the differentiation of Purkinje cells in the cerebellum was strongly retarded [39]. As a result, we can suspect that excessive thyroid hormones in thyrotoxicosis have impact on the neurons within cerebellum and gradually generate certain cognitive and emotional impairments.

Additionally, hyperthyroid patients displayed significantly decreased DC in bilateral MeFG, and we also discovered abnormal FC of seed-2 located in bilateral MeFG to the left IOG, caudate and DMN regions including bilateral PCu and bilateral MeFG. Abnormal functional connectivity of DMN was reported to contribute to disrupted working memory [40, 41] and emotional processing [42–44]. Previous studies of hyperthyroidism have revealed impaired metabolism and FC in brain regions anchored in DMN [7–9, 12, 13]. The related brain regions of DMN in hyperthyroidism primarily included PCC [8, 9, 12], inferior frontal gyrus [13] and superior frontal gyrus [7]. In the current study, we also found aberrant DMN functional connectivity in MeFG and PCu, and the FC between bilateral MeFG and PCu was negatively correlated with the processing speed Z scores. This implicated a vital role of DMN in regulating hyperthyroid patients’ cognitive and mental function. In addition to the internal abnormalities within DMN, the interaction between DMN and other association brain networks also played an important role in modulating cognition and mood. For example, we found the disrupted resting-state FC between DMN and cerebellum mentioned above. Moreover, decreased FC between DMN and left IOG in visual network was also detected in this current study. The IOG resided in the primary visual cortex and appeared to be involved in processing visual recognition [45, 46]. A study by Golby et al. [47] suggested that altered activation in occipital gyrus was correlated with impaired visual memory of Alzheimer's disease. As a result, the reduced functional connectivity between DMN and occipital gyrus may result in the damage of visuospatial skills in hyperthyroidism. Prior studies about obsessive-compulsive disorder provided evidence that damaged functional connectivity between caudate nucleus, ACC and prefrontal cortex within the cortico-striato-thalamo-cortical circuitry possibly caused the impaired cognitive and behavioral control in obsessive-compulsive patients [48–50]. Thus, attenuated functional connectivity between bilateral MeFG of DMN and left caudate in hyperthyroid group is likely to compose the pathophysiology of cognitive and emotional deficits, and the negative correlation between FT3 levels and MeFG-PCu functional connectivity further demonstrated the importance of DMN. In short, the DMN played a key role in facilitating the process of the exasperation of neuropsychological performance in hyperthyroidism.

Degree centrality analysis is not firstly applied to hyperthyroidism. One literature by Göttlich et al. [13] used to compute DC value in hyperthyroid patients, but totally different results can be found between the former report and our study. The previous study has declared significantly increased DC in rostral temporal lobes and increased FC between temporal poles and the cognitive control network [13]. While our study has illuminated obviously decreased DC mainly in the left PLC and left MeFG. The possible reasons for the difference may derive from the various data processing method and chosen population. However, the crucial cause for it lies in the selected patients in the two studies. The prior research recruited healthy men receiving 8 weeks’ oral administration of 250 μg levothyroxine per day, thus the hyperthyroid patients all got drug-induced hyperthyroidism. On the contrary, the hyperthyroid subjects (26 females and 7 males) enrolled in our study were all diagnosed as Graves’ disease. Graves’ disease is a kind of autoimmune thyroid disorder by stimulating antibodies to the TSH receptor on thyroid follicular cells. It is featured with excessive FT3, FT4, thyrotropin receptor antibodies (TRAb) and suppressed TSH. Besides, most serum TPOAb levels elevate in Graves’ disease and are reported to be correlated with neuropsychiatric performances. Yu et al. [51] yielded a study showing that high serum TPOAb level in subclinical hypothyroidism after I-treatment of Graves’ disease was an independent risk factor for depression. And TPOAb was also proved to be associated with abnormal neuropsychiatric symptoms in autoimmune thyroid diseases, including Hashimoto's thyroiditis [52] and Hashimoto's encephalopathy [53–55]. The possible mechanisms proposed included autoimmune central nervous system (CNS) vasculitis with or without immune complex deposits, and autoimmune reaction to antigens shared by the thyroid gland and the CNS [56]. In this current study, the TPOAb level was negatively correlated with DC value in the left PLC, implying the importance of TPOAb in Graves’ disease. Therefore, our study differs greatly from the pre-existing study because of the etiology of hyperthyroidism. Our study emphasizes on Graves’ disease-induced hyperthyroidism, a kind of autoimmune disorder, while the other one is drug-induced hyperthyroidism.

This study also has a few limitations. Firstly, this is a cross-sectional study, whether the altered functional connectivity is reversible after therapy remains to be discussed by the prospective study. Secondly, the trier being unable to control the participants’ thoughts during imaging is a common problem to resting-state studies. Although participants were instructed not to move their heads and to rest with their eyes closed, slight head movements and rotation are unavoidable. However, we have inspected each image carefully, and patients with head movements greater than 2.5° or 2.5 cm were excluded. Besides, on account of laboratory testing limiting in hospital, exact Figures of FT4, TSH and TRAb were unreachable, so we could not compare the variance of brain activation in hyperthyroidism of different severity. Finally, patients recruited in this study were suffering from Graves’ disease-induced hyperthyroidism, which thus made it possible that the functional connectivity might result from the autoimmune response rather than the thyroid hormones. In the future study, we will add another control group of autoimmune disorder to Figure out whether the autoimmune response leads to the changes in Graves’ disease. Given these limitations, future studies should be well designed, taking these results into consideration. Future fMRI studies could investigate these patients after recovery from hyperthyroidism.

In conclusion, the decreased intrinsic functional connectivity in the left PLC and MeFG, as well as the abnormal resting-state FC anchored in DMN, attention network, visual network and cognitive network, constitutes the underlying mechanism for neuropsychological changes in hyperthyroidism. The findings of this study suggest a better understanding of the nature of disconnection in hyperthyroid patients, which might be helpful to figure out the neurobiological mechanism of emotional and cognitive impairments of hyperthyroidism.

## MATERIALS AND METHODS

### Subjects

A total of thirty-three right-handed hyperthyroid patients and thirty-three age-, sex- and education-matched healthy controls were recruited (age range: 18–60 years, education range: 9–22 years). All the patients had elevated serum FT3, FT4, TRAb levels, and inhibited TSH levels. However, the thyroid hormones of the control group were within normal ranges (FT3 1.8–4.6 pg/ml, FT4 0.93–1.7 ng/dl, TRAb 0–1.75 IU/L, TSH 0.27–4.2μIU/ml). Exclusion criteria for all the participants were as follows: (1) A history of neurological or psychiatric illness, including head injury, cerebral hemorrhage, major depression and so on; (2) Drug or alcohol abuse history; (3) A history of cardiovascular or pulmonary diseases that affected BOLD fluctuations; (4) Contraindications to MRI scanning; (5) Head motion more than 1.0 mm or 1.0 u during MRI scanning. The thyroid hormone levels, disease duration, height, weight and family history were recorded in this research. This study was approved by the Medical Ethics Committee for Clinical Research of Zhongda Hospital Affiliated to Southeast University. All the participants signed written informed consent prior to the study.

### Neuropsychological assessments

A series of psychometric and domain-specific cognitive tests were conducted for each subject. The mood tests included Hamilton depression rating scale 17 (HDRS-17) and the Hamilton anxiety rating Scale 17 (HARS-17). The domains of cognition tests consisted of processing speed, executive function, visuospatial skills and episodic memory. Processing speed was evaluated by portions of the Trail Making Test (TMT A), Digit Symbol Substitution Test (DSST), the Stroop Color and Word Test (Stroop Color and Stroop Word). Executive function was assessed via Verbal Fluency Test (VFT-animal, VFT-verb), the Stroop Color Word Test (Stroop Inhibition), TMT B and Digit Span Test (DST). Visuospatial Skills were estimated with Rey-Osterrieth Complex Test (CFT) and Clock Drawing Test (CDT). Episodic memory was assessed with delayed recall of Auditory Verbal Learning Test (AVLT-DR) and Rey-Osterrieth Complex Test (CFT-DR). Two board-certified clinical psychiatrists scored these tests.

### MRI data acquisition and date analysis

### MRI data acquisition

All imaging data were acquired using a 3.0T MRI scanner (Siemens MAGNETOM Trio, Erlangen, Germany) with a standard head coil. All subjects were instructed to lie quietly with head fixed by a belt and ears covered with foam padding and earplugs in order to reduce head motion and scanner noise. Meanwhile, they were required to close their eyes but remain awake, and avoid specific thoughts during the scan. Resting state images were acquired using a gradient-echo planar sequence with the following scan parameters: slices = 36, repetition time = 2000 ms; echo time = 25 ms; flip angle = 90°; acquisition matrix = 64 × 64; field of view = 240 mm × 240 mm; thickness = 3.0 mm; gap = 0 mm and 3.75 mm × 3.75 mm in-plane resolution parallel to the anterior commissure–posterior commissure line. For each participant, fMRI scanning lasted 8 minutes and 240 volumes were obtained.

### Image preprocessing

Data preprocessing was conducted using Data Processing Assistant for rs-fMRI (DPARSFA) software. The first ten volumes were discarded because of adaptation of participants to the scanning noise and spin saturation. Slice timing and realignment for head motion correction were performed. Subjects with a head motion > 2.5mm translation or a 2.5° rotation in any direction were excluded. After that, we normalized the images into a standard stereotaxic space using a 12-parameter affine approach and an EPT template image that was resampled to 3mm × 3mm × 3mm voxels. Then spatial smoothing was conducted with a Gaussian kernel of 6mm full width at half maximum to reduce spatial noise. Ultimately, removal of linear trends and band-pass filtering (0.01Hz < f < 0.08Hz) were performed to reduce low-frequency drift and high-frequency physiological noise.

### DC calculation

DC attributes a greater value to a voxel if it has strong connections with many other voxels in the brain. For the calculation of voxel-wise DC, the preprocessed fMRI data were used to compute the voxel-based whole-brain functional correlation analysis according to the method that has well been described in previous studies [17, 57]. The Pearson's correlation coefficient (r) between each pairs of brain gray matter voxels were computed. As a result, we acquired a matrix of Pearson correlation coefficients depicting whole-brain functional connectivity pattern. To obtain each subject's graph, whole-brain functional network was then constructed by defining threshold of each correlation at r > 0.25 [17, 18]. The threshold is the default setting in the calculation of the DC map and was chosen to remove voxels that had low temporal correlation attributable to signal noise. Finally, the voxel-wise DC values were converted into a Z-score map by the Fisher-Z transformation to improve normality. Brain regions showing high z-scores are deemed as hub regions which are highly connected and thus play a vital role in the network integration. As previously reported, given the negative correlations’ uncertain interpretation and detrimental effects on test-retest reliability, only positive correlations were considered in the DC calculations.

### Seed-based connectivity analyses

Seed-based connectivity analyses were conducted to investigate the functional connectivity of regions with abnormal DC to the whole brain. Seeds were constructed by drawing a 6-mm radius sphere around the center voxels of these regions, and the time series for every seed was extracted from the preprocessed data. Time series were averaged across all voxels within each seed's sphere. Pearson's correlation analyses were conducted between the seeds and the remaining voxels. The resulting r values were transformed to Z values to improve the Gaussianity of their distribution. Resting-state FC analysis can specifically provide aberrant functional connectivity pattern of each seed.

### Statistics analysis

We analyzed the differences in demographic and neuropsychological performances between hyperthyroid patients and healthy controls by various statistical methods. Two-sample *t*-tests and Mann-Whitney rank tests were used for continuous variables, and Chi-square test was applied for categoric variables (statistical significance was set at P < 0.05) by SPSS 21.0 software (SPSS, Inc., Chicago, IL).

We divided the cognitive tests into 4 domains as above (ie, processing speed, executive function, visuospatial skills and episodic memory). In order to combine the cognitive variables, the standardized Z scores of each individual test, which were created by using control group data across all patients, were summed to figure out the cognitive domain values. Variables in which good performance was represented by lower values (e.g. TMT, Stroop color and Stroop word) were adjusted for reciprocal transformation to ensure that higher Z-scores represented better performance for all variables. Independent sample *t*-test was performed to compare the mean Z-scores for each neuropsychological test and cognitive domain with in order to compare the patterns of neurocognitive impairments between the two subgroups.

To investigate significant differences of DC and resting-state FC between hyperthyroidism and heathy controls, a two-sample *t*-test was performed in a voxel wise manner by REST software [58]. The result was corrected for multiple comparisons using the AlphaSim program based on Monte Carlo simulation algorithm. The statistical threshold for DC was set at P < 0.05 and cluster size > 143 voxels. Meanwhile the threshold for resting-state FC was set at a corrected P < 0.001 and cluster size > 22 voxels. The thresholds were obtained with RSET software. Then, within the hyperthyroidism group, partial correlation analyses were applied to examine relationships between DC, FC values and both standardized neuropsychological performance scores as well as some clinical indexes by SPSS 21.0 software, controlling for age, gender and duration of education as confounding variables.

## SUPPLEMENTARY MATERIALS FIGURES AND TABLES



## Abbreviations

MNI: Montreal Neurological Institute space
HPs: hyperthyroid patients
HCs: health controls
BA: Brodmann area
MTG: middle temporal gyrus
PLC: posterior lobe of cerebellum
IOG: inferior occipital gyrus
PCu: precuneus
MeFG: medial frontal gyrus.
