# Supplementary material for: Abnormal brain functional connectivity leads to impaired mood and cognition in hyperthyroidism: a resting-state functional MRI study
Source: Oncotarget. 2016 Dec 21;8(4):6283–94. doi: 10.18632/oncotarget.14060 (PMC5351631; doi:10.18632/oncotarget.14060)
Supplement: Supplementary file 1 [file oncotarget-08-6283-s001.pdf]

# Abnormal brain functional connectivity leads to impaired mood and cognition in hyperthyroidism: a resting-state functional MRI study

## Supplementary Materials

### Statistical analysis

To increase statistical power by reducing random variability, a composite score analysis was utilized, as previously introduced [1]. First, the raw scores from each test for each subject were transformed to z scores with reference to the means and standard deviations of each test for all subjects. Second, all neuropsychological tests were grouped into four cognitive domains and the related composite scores were calculated by averaging the z scores of the individual tests according to the following divisions: Episodic Memory (two tests, including AVLT-DR, CFT-DR), Visuospatial Skills (two tests, including CFT, CDT), Processing Speed (three tests, including Stroop Color, Stroop Word, TMT-A, DSST), and Executive Function (five tests, including Stroop Inhibition, TMT-B, DST, VFT-1, VFT-2).

## REFERENCE

1. Sexton CE, McDermott L, Kalu UG, Herrmann LL, Bradley KM, Allan CL, Le Masurier M, Mackay CE, Ebmeier KP. Exploring the pattern and neural correlates of neuropsychological impairment in late-life depression. Psychol Med. 2012; 42:1195–1202.

## Supplementary Table S1: Raw scores of neuropsychological tests

| Characteristic | Hyperthyroidism group (*n* = 33) | Control group (*n* = 33) | *p*-value |
|---|---|---|---|
| **Neuropsychological test data (z-score)** | | | |
| **Mood** | | | |
| **HDRS** | 8.58 ± 5.23 | 1.03 ± 1.33 | < 0.001[a] |
| **HARS** | 9.39 ± 4.58 | 1.00 ± 1.22 | < 0.001[a] |
| **Processing Speed** | | | |
| **Stroop Color (second)** | 37.09 ± 12.99 | 32.61 ± 7.23 | 0.089[a] |
| **Stroop Word (second)** | 23.33 ± 6.70 | 21.18 ± 5.37 | 0.155[a] |
| **TMT-A (second)** | 52.18 ± 23.36 | 43.36 ± 15.63 | 0.077[a] |
| **DSST** | 56.58 ± 17.87 | 62.64 ± 17.54 | 0.169[a] |
| **Executive Function** | | | |
| **Stroop Inhibition (second)** | 61.79 ± 16.74 | 61.64 ± 18.73 | 0.972[a] |
| **TMT-B (second)** | 107.67 ± 41.75 | 89.61 ± 31.84 | 0.049[a] |
| **DST** | 12.15 ± 2.51 | 13.67 ± 2.38 | 0.014[a] |
| **VFT-1** | 20.55 ± 6.26 | 24.70 ± 5.33 | 0.005[a] |
| **VFT-2** | 25.24 ± 8.47 | 29.33 ± 5.82 | 0.026[a] |
| **Visuospatial Skills** | | | |
| **CFT** | 33.39 ± 3.67 | 35.33 ± 1.29 | 0.007[a] |
| **CDT** | 8.24 ± 1.28 | 9.58 ± 0.97 | < 0.001[a] |
| **Episodic Memory** | | | |
| **AVLT-DR** | 7.46 ± 2.55 | 7.79 ± 2.75 | 0.611[a] |
| **CFT-DR** | 18.06 ± 8.22 | 23.00 ± 7.14 | 0.011[a] |

Abbreviations: HDRS = Hamilton Depression Rating Scale, HARS = Hamilton Anxiety Rating Scale, Stroop Color Total Time = Color naming subtest scaled score, Stroop Word Total Time = Word naming subtest scaled score, Stroop Inhibition Time = inhibition subtest scaled score; TMT = Trail-Making Test, DSST = Digit Symbol Substitution Test, DST = digit span test, VFT =Verbal Fluency Test, CDT = clock-drawing test, CFT = Rey-Osterrieth complex figure test, CFT-DR = Rey-Osterrieth complex figure test delayed recall, AVLT-DR = auditory verbal learning test–delayed recall.

[a]Two independent sample *t*-test.
